# The Sociodemographic and Risk Factors for Fuchs’ Endothelial Dystrophy: A Nationwide, Matched Case–Control Study in Taiwan

**DOI:** 10.3390/jpm12020305

**Published:** 2022-02-18

**Authors:** Yuh-Shin Chang, Chung-Han Ho, Jhi-Joung Wang, Sung-Huei Tseng, Ren-Long Jan

**Affiliations:** 1 Department of Ophthalmology, Chi Mei Medical Center, Tainan 710, Taiwan; yuhshinchang@yahoo.com.tw (Y.-S.C.); shtseng1@gmail.com (S.-H.T.); 2 Graduate Institute of Medical Sciences, College of Health Sciences, Chang Jung Christian University, Tainan 711, Taiwan; 3 Department of Medical Research, Chi Mei Medical Center, Tainan 710, Taiwan; ho.c.hank@gmail.com (C.-H.H.); 400002@mail.chimei.org.tw (J.-J.W.); 4 Department of Hospital and Health Care Administration, Chia Nan University of Pharmacy and Science, Tainan 717, Taiwan; 5 Department of Anesthesiology, Chi Mei Medical Center, Tainan 710, Taiwan; 6 Department of Ophthalmology, National Cheng Kung University Hospital, College of Medicine, National Cheng Kung University, Tainan 701, Taiwan; 7 Department of Pediatrics, Chi Mei Medical Center, Liouying, Tainan 736, Taiwan

**Keywords:** Fuchs’ endothelial dystrophy, ocular allergic conditions, case–control study, sociodemographic factors, Taiwan Longitudinal Health Insurance Database

## Abstract

This nationwide, population-based, retrospective, matched case–control study included 4334 newly diagnosed Fuchs’ endothelial dystrophy (FED) patients who were identified by the International Classification of Diseases, Ninth Revision, Clinical Modification (ICD-9-CM), code 371.57, and selected from the Taiwan National Health Insurance Research Database. The age-, sex-, and index-date-matched control group included 4334 non-FED controls selected from the Taiwan Longitudinal Health Insurance Database 2000. Ocular allergic conditions and sociodemographic conditions were examined using univariate logistic regression analyses and paired *t*-test was used for continuous variables. Adjusted logistic regression was used to compare the odds ratio (OR) of the FED development. Patients with ocular allergic conditions were more likely to have FED than the controls (OR = 25.50, 95% CI = 12.58–51.68, *p* < 0.0001) even after conditional logistic regression was conducted (adjusted OR = 25.26, 95% CI = 11.24–56.77, *p* < 0.0001). Regarding the sociodemographic factors, we found that more than half of the FED patients in Taiwan were aged ≥45 years old, there was an equal female-to-male ratio (1.06:1), and patients with a lower income and living in northern Taiwan had higher odds of developing FED. The results strongly support an association between ocular allergic conditions, geographic region, residential status, income, and FED.

## 1. Introduction 

Fuchs’ corneal endothelial dystrophy (FED), the most common form of corneal dystrophy, affects the endothelium, which is the innermost layer of the cornea. FED is characterized by endothelial cell density reduction with endothelium cell morphology alterations including variation in cell shape, known as cellular pleomorphism, and variation in the cell size, known as polymegathism [1]. FED usually presents in the fifth decade of life and progresses over the next two to three decades with continued endothelium cell loss and dysfunction. Some FED patients may be asymptomatic in the early stages of the disease, but patients may have glare or reduced visual acuity, severe pain due to the corneal edema progression to stromal thickness, increased bulla formation, or even long-standing corneal vascularization [2].

FED is a multifactorial disorder caused by a complex combination of genetic, biochemistry, biology, and environmental factors. The pathophysiology of FED remains unknown, although several proposed mechanisms have been reported [1,3,4,5]. Channelopathy, related to mutations in the genes of the ion channels in the corneal endothelium appears to be an important pathogenetic factor in the development of FED [3,4]. Elevation of oxidative stress and reactive oxygen species accumulation could lead to apoptosis of endothelial cells and is also regarded as one major cause of the development of FED [1,4]. The epithelial–mesenchymal transition, in which fibroblastic or epithelial cell phenotypes transform from endothelial cells, could result in the secretion of extracellular matrix proteins leading to abnormal deposition, is thought to be involved in the pathogenesis of FED [1,4,5].

The estimated incidence and prevalence of FED varies greatly worldwide, with a higher prevalence in Europe and the USA and lower rates in Asia, possibly because of different genetic or environmental factors and a difference in clinical definitions of FED [6,7]. Being older than 40 years of age is a major risk factor for FED development [4,5,8]. Several studies have reported that FED is more predominant in the female sex, with an increased odds ratio (OR) [5,9]. However, patients with diabetes mellitus (DM), which is known to influence endothelial cell degeneration and dysfunction, were not more likely to have FED [10]. The conflicting evidence in the pathophysiologic theory and the clinical evaluation means that further clarification is required between the association of DM and the development of FED. 

It is important to note that in the present study, we evaluated the association between atopic characteristics including ocular allergic conditions (allergic conjunctivitis, atopic keratoconjunctivitis, and vernal keratoconjunctivitis), asthma, allergic rhinitis, and atopic dermatitis based on the proposed pathophysiologic mechanisms of FED [1,3,4,5]. The oxidative stress that results in endothelium apoptosis and extracellular matrix protein remodeling might be triggered by these atopic conditions, especially after eye rubbing in patients with ocular allergic conditions [11,12,13,14].

The purpose of the current study was to use a health care claims database containing records for more than 4000 FED patients and controls matched by age and sex to investigate the association between ocular allergic conditions, sociodemographic factors, various comorbid conditions, and FED, which may help to elucidate the pathophysiologic features of FED.

## 2. Materials and Methods

### 2.1. Database 

The data for our cohort study were taken from the National Health Insurance Research Database (NHIRD), provided by the National Health Research Institute (NHRI), Taiwan. The NHIRD provides encrypted patient identification numbers together with information on patient demographics such as date of birth, sex, dates of admission and discharge, and residential area. It also incorporates the International Classification of Diseases, Ninth Revision, Clinical Modification (ICD-9-CM) codes, which records diagnoses and procedures, prescription items, as well as costs covered by the NHI. The research was exempt from review by the Institutional Review Board of the Chi Mei Medical Center.

### 2.2. Selection of Patients and Variables

**Figure 1:**
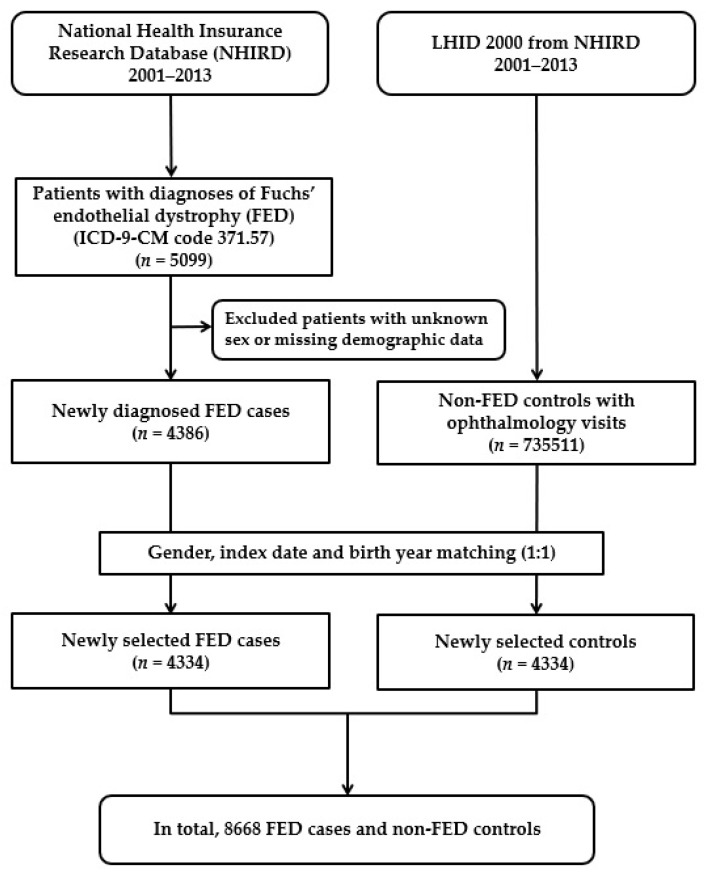
Flowchart demonstrating the enrollment of patients with Fuchs’ endothelial dystrophy (FED) and the control patients.

A newly diagnosed FED group and a matched non-FED control group were enrolled in this population-based case–control study. The patient information from both groups was collected from 1 January 2001 to 31 December 2013, and both groups were traced until the end of 2013. A flowchart of our study is shown in Figure 1. Initially, 5099 patients with a diagnosis of FED (ICD-9-CM code 371.57) were included. In total, 4386 patients with newly diagnosed FED were enrolled after we excluded patients with unknown sex or missing demographic data. Finally, there were 4334 patients with a diagnosis of FED taken from the NHIRD after matching with the control. For each patient with FED, one non-FED control was randomly chosen from the Longitudinal Health Insurance Database 2000 (LHID 2000), which is a subset of the NHIRD and contains the overall claim data for one million beneficiaries for the year 2000. Initially, we included 735,511 subjects who had at least one ophthalmology visit and no FED diagnosis, before the index date, from the one million subjects of the LHID 2000, after excluding patients with missing sex or demographic data. In total, controls (*n* = 4334) were matched with FED patients via propensity scores by age (±30 days), sex, and index date, which was defined as the first day of diagnosis with FED. Each participant in both groups was tracked, and the demographic data of each participant were recorded from the index date until the end of 2013 or death, whichever was earlier. To determine the medical comorbidities for FED, data regarding comorbid conditions such as ocular allergic conditions (allergic conjunctivitis (ICD-9-CM code 372.14), atopic keratoconjunctivitis (ICD-9-CM code 372.05), and vernal keratoconjunctivitis (ICD-9-CM code 372.13, 370.32)), asthma (ICD-9-CM code 477), allergic rhinitis (ICD-9-CM code 493), atopic dermatitis (ICD-9-CM code 691), DM (ICD-9-CM code 250), chronic renal disease (ICD-9-CM code 582–588 except 584 and 587), and mitral valve prolapses (ICD-9-CM code 424.0) were collected. These comorbidities were identified based on an ICD-9-CM code recorded within one year before the index date and ascertained by three or more ambulatory care claims or admittance as an inpatient.

### 2.3. Statistical Analysis

All statistical analyses were performed using SAS 9.4 for Windows (SAS Institute, Inc., Cary, NC, USA). Demographic characteristics such as age group, sex, income, geographic region, residential city status, and occupation were analyzed using McNemar’s test, and continuous variables were calculated using the paired *t*-test. In addition, comorbid conditions (ocular allergic conditions, asthma, allergic rhinitis, atopic dermatitis, DM, chronic renal disease, and mitral valve prolapses) were compared between FED patients and controls using McNemar’s test. ORs obtained by univariate logistic regression analyses and a multivariable logistic regression model (conditional on age, sex, and index date) were constructed to compute the adjusted OR of various comorbidities with a diagnosis of FED. The independent variables included sociodemographic factors (income, geographic region, residential city status, occupation, and the ophthalmology visit times within one year before the index date), all the abovementioned medical conditions of interest. The level of significance was set at *p* < 0.05.

## 3. Results

### 3.1. Demographic Data

After ineligible patients were excluded, 4334 FED patients and 4334 age- and sex-matched controls who had used medical care services covered by the NHI between 2004 and 2011 were analyzed. The mean ages of the FED patients and the controls were 49.78 (standard deviation (SD) 17.83) and 49.80 (SD 17.81) years, respectively (Table 1). Of the 4334 FED patients, 366 (8.44%) were younger than 25 years, 625 (14.42%) were aged 25 to 34 years, 780 (18.00%) were aged 35 to 44 years, and 2563 (59.14%) were aged 45 years or over. Among the 4334 FED patients, 2105 (48.57%) were men and 2229 (51.43%) were women. The incomes of FED patients were significantly different from the controls. The most common approximate income of FED patients was lower than 30,000 New Taiwan dollars (TWD) (2523; 58.21%), followed by between TWD 30,000 and 60,000 (1507; 34.77%); between TWD 60,000 and 90,000 (242; 5.58%); higher than TWD 90,000 (62; 1.43%) (*p* = 0.0124). With regard to geographic distribution, the most common region of residence of the patients diagnosed with FED was northern Taiwan (3574; 81.84%), followed by the southern (433; 9.99%), central (305; 7.04%), and eastern regions (49; 1.13%), with a significant difference from the controls (*p* < 0.0001). Most FED patients resided in a metropolis city (3703; 85.44%), compared with those in a satellite city (148; 3.41%) and rural areas (483; 11.14%), with a significant difference from the controls (*p* < 0.0001). With regard to occupation classification, a significant difference in the distribution was found between the two groups, with over half of the 4334 FED patients being public servants, including military, civil, or teaching staff (2460; 56.76%); the remaining patients were farmers (298; 6.88%) and fishermen (42; 0.97%) (*p* < 0.0001). It is worth noting that patients with FED visited an ophthalmologist more than twice the number of times within one year before the index date (17.00 ± 28.70) than the control group (6.53 ± 12.86; *p* < 0.0001). The FED patients exhibited a significantly higher prevalence of ocular allergic conditions (204; 4.71%) than the controls (*p* < 0.0001). There were no significant differences regarding other possible comorbidities such as asthma, allergic rhinitis, atopic dermatitis, chronic renal disease, and mitral valve prolapse between FED patients and controls. A significantly lower prevalence of DM in FED patients (310; 7.15%) than in the controls (428; 9.88) (*p* < 0.0001) was noted, although DM was a previously reported comorbidity.

### 3.2. Associated Risk Factors

Sociodemographic factors including income, geographic region, residential city status, and occupation of the FED patients and controls were examined using univariate logistic regression analyses and a multiple logistic regression model with adjustments for age, sex, sociodemographic factors, and comorbidities. Patients whose income was between TWD 30,000–60,000 had increased odds of developing FED relative to those with an income > TWD 90,000 (OR = 1.45, 95% confidence interval CI = 1.04–2.04, *p* *=* 0.0302). An income < TWD 30,000 and between TWD 30,000–60,000 continued to be a significant risk factor for FED after adjustment for other confounders (adjusted OR = 1.92, 95% CI = 1.28–2.87, *p* = 0.0016; adjusted OR = 1.72, 95% CI = 1.16–2.56, *p* = 0.0074, respectively). Regarding the geographic location and the residential city status of the patient’s residence, patients who lived in northern Taiwan or a metropolis city showed a significantly higher prevalence of FED (OR = 4.80, 95% CI = 3.37–6.83, *p* < 0.0001; OR = 2.53, 95% CI = 2.23–2.88, *p* < 0.0001, respectively) relative to those who lived in eastern Taiwan or a rural area, and who lived in northern Taiwan remained a significant risk factor after a conditional logistic regression analysis was conducted (adjusted OR = 5.33, 95% CI = 3.42–8.30, *p* < 0.0001). 

Several possible comorbidities were also examined using univariate and multiple logistic regression analyses (Table 2). Patients with ocular allergic conditions had significantly higher ORs of receiving a diagnosis of FED (OR = 25.50, 95% CI = 12.58–51.68, *p* < 0.0001) even after conditional logistic regression was conducted (adjusted OR = 25.26, 95% CI = 11.24–56.77, *p* < 0.0001). Patients with asthma, allergic rhinitis, atopic dermatitis, chronic renal disease, and mitral valve prolapse did not have higher ORs of receiving a FED diagnosis. DM patients had reduced odds of a FED diagnosis before and after adjustment for other confounders (OR = 0.68, 95% CI = 0.58–0.80, *p* < 0.0001; adjusted OR = 0.52, 95% CI = 0.42–0.64, *p* < 0.0001).

## 4. Discussion

To the best of our knowledge, this study appears to be the largest nationwide, population-based, case–control study to evaluate the association between ocular allergic conditions, sociodemographic factors, common comorbid conditions, and FED. These analyses identified several key findings. First, more than half of the FED patients in Taiwan were aged ≥45 years old, and there was an equal female-to-male ratio (1.06:1). Second, the odds of developing FED varied with several sociodemographic factors. Patients with a lower income and living in either northern Taiwan or a metropolis city had higher odds of developing FED. Third, some comorbid conditions significantly influenced the odds of developing FED. By comparison, patients with ocular allergic conditions had considerably higher odds of developing FED. We observed that DM patients were less likely to have FED, compared with non-DM subjects.

We were unable to study whether age and sex affected the risk of developing FED after matching these demographic factors between cases and controls; in addition, we found that, of the 4334 FED patients, 2563 (59.14%) were older than 45 years. This finding was consistent with that reported in many previous reports [2,4,15]. The reasons for why there was a higher prevalence of FED in patients aged ≥45 years old in the current study are based on the following two points: Firstly, it was found that corneal endothelium degeneration including cell density reduction, together with cell morphology alteration, correlated with increasing age [15]. Secondly, in the early stages, FED patients may be asymptomatic until the endothelium cell numbers have decreased to a level that no longer maintains corneal clarity, resulting in reduced vision and/or eye pain [2].

FED patients did not demonstrate a female preponderance in the current study, which contradicts earlier investigations, in which FED was reported to have a female-to-male ratio of 2.5:1 to 3.5:1 [9,15,16,17]. FED showed a female preponderance with a 3.5:1 ratio in patients who underwent penetrating keratoplasty due to FED at Duke University Eye Centre, USA [9]. Increased prevalence of FED in females was observed in other developed countries such as Iceland (11% females vs. 7% males), Singapore (8.5% females vs. 4.4% males), and Japan (5.5% females vs. 1.5% males) [15,16]. The inconsistency of the higher FED prevalence in females between our results and the previous reports may be related to the differences in socioeconomic conditions. It may be possible that the increased incidence of FED in females is only observed in developed countries. The Taiwanese population did not demonstrate female predominance in FED prevalence; this may be because Taiwan is a developing country. Other possible explanations of this inconsistency may be associated with racial, geographic location, environmental factors, and even perhaps reflecting differences in underlying pathogenic mechanisms such as genetic predisposition that may affect the susceptibility of the female sex to FED; such differences need to be clarified by more local epidemiologic FED studies in the future.

Regarding the sociodemographic factors, we found statistically significant associations between FED and patients living in northern Taiwan. The higher rate of FED diagnosis in northern Taiwan in our study may reflect the availability of low-cost medical care, ease of visits to ophthalmologists, and enhanced access to corneal specialists for diagnosis and management of FED, compared with other regions of Taiwan. Individuals with a lower income had significantly higher odds of developing FED. This is possible that FED patients would have limitations in employment due to limited visual acuity from the disease.

In the current study, patients with ocular allergic conditions had a remarkably higher OR for FED development (adjusted OR = 25.26, 95% CI = 11.24–56.77, *p* < 0.0001). Possible explanations for this finding include the observation that chronic habitual eye rubbing is a common manifestation in patients with ocular allergic conditions [14]. Elevation in oxidative stress following eye rubbing could trigger reactive oxygen species accumulation and endothelium apoptosis, which is regarded as an important pathophysiologic factor for FED formation [4,14,18,19]. It is also possible that there is an increased expression in extracellular matrix proteins such as collagen and gelatin after inflammatory events related to eye rubbing in patients with ocular allergic conditions [12,13]. The upregulation of extracellular matrix proteins and their subsequent deposition leads to the thickening of Descemet’s membrane, which may play a role in the pathophysiology of FED development [1,4,5]. To the best of our knowledge, no previous study has shown the association between ocular allergic conditions and FED development. The association between ocular allergic conditions and FED highlighted in the current study should be clarified in future studies, to enhance the understanding of the pathophysiology of FED.

Although DM has been reported to cause endothelial cell death and dysfunction and is expected to increase the risk of FED development, there has been no conclusion as to whether DM is a risk factor for FED [10,20,21,22]. Further, in the study by Zhang et al., production of free radicals increased, and antioxidant defense capabilities were impaired in DM patients; however, DM was not linked to an increased risk of development of FED [5]. In the current study, we have to highlight a contradiction—namely, that DM has a negative effect on the corneal endothelium but, at the same time, is a significant protective factor of FED. This confliction is hypothesized to be explained by the fact that more ocular complications occur in DM patients including corneal erosion, corneal epithelium defect, and even bullous change, which might mask the diagnosis of FED. However, we also propose that death censoring might have played a role in the DM patients as the proportion of DM patients who died before FED progression might be higher than non-DM subjects.

The current study had several strengths. To the best of our knowledge, our study is the largest to date to focus on FED patients, with more than 4000 cases identified in the NHIRD database. These analyses identified FED patients throughout the country who had received a diagnosis from a comprehensive ophthalmology exam at an array of different hospitals, including clinics, district hospitals, regional hospitals, and medical centers. The selection bias regarding referral centers was reduced because the data from our study were based on a nationwide and population-based dataset. The recall bias was obviated because the claims data of the NHIRD were recorded electronically and did not rely on the patient self-reporting their medical conditions. In addition, our study was case–control and incorporated 10 years of longitudinal data on various sociodemographic factors, ocular allergic conditions, and comorbid conditions in FED patients and controls. Our results are reliable, because these sociodemographic factors, ocular allergic conditions, and comorbid conditions were recognized as potential confounding factors when assessing the OR in FED patients, and appropriate adjustments were made for these potentially confounding variables.

This study had several limitations. First, the presence of FED among those identified as patients or the absence of FED in the controls could not be confirmed based on the claims data, without access to clinical records. In addition, the diagnosis of FED and other comorbid disorders was based on ICD-9-CM codes, which may lead to disease misclassification. Second, because the medical history of the patients sampled could only be traced back to 1996, there was no information to confirm that the controls had not been diagnosed with FED before January 1996, potentially compromising our findings. In addition, the significantly higher number of ophthalmology visits in the FED group, compared with the control group, may have resulted in the FED patients having a higher chance of being diagnosed with ocular allergic conditions, which could have resulted in a surveillance bias in our study. To reduce the surveillance bias, we added the ophthalmology visit times within one year before the index date as an important confounder. Finally, the patients and controls personal lifestyles that might contribute to FED, such as smoking or the amount of UV light exposure, were not available in the administrative database, potentially compromising our results.

## 5. Conclusions

In summary, the current study identified several sociodemographic factors that were associated with an increased risk of FED including living in northern Taiwan and a lower income. It is important to note that, after controlling for sociodemographic factors and possible comorbidities such as DM, chronic renal disease, mitral valve prolapse, asthma, allergic rhinitis, and atopic dermatitis, patients with ocular allergic conditions had a remarkably and significantly higher risk of developing FED than the controls. This association might be helpful to clarify and enhance the understanding of the pathophysiology of FED.

## Figures and Tables

**Table 1:** Baseline sociodemographic factors and comorbid conditions of Fuchs’ endothelial dystrophy patients and control subjects after propensity score matching by age and gender.

	Fuchs’ Endothelial Dystrophy *n* = 4334	Comparison *n* = 4334	*p* Value
Sociodemographic factors	*n* (%)	*n* (%)	
Age (year; Mean ± SD)	49.80 ± 17.83	49.78 ± 17.81	0.9988 ^a^
Age (year)			
<25	366 (8.44)	366 (8.44)	1.000 ^b^
25–34	625 (14.42)	625 (14.42)	
35–44	780 (18.00)	780 (18.00)	
45–54	875 (20.19)	875 (20.19)	
55–64	736 (16.98)	736 (16.98)	
≥65	952 (21.97)	952 (21.97)	
Gender			
Male	2105 (48.57)	2105 (48.57)	1.000 ^b^
Female	2229 (51.43)	2229 (51.43)	
Income			0.0124 ^b^
<TWD 30,000	2523 (58.21)	2620 (60.45)	
TWD 30,000–60,000	1507 (34.77)	1378 (31.80)	
TWD 60,000–90,000	242 (5.58)	253 (5.84)	
>TWD 90,000	62 (1.37)	83 (1.91)	
Geographical region of Taiwan			<0.0001 ^b^
Northern	3547 (81.84)	2225 (51.34)	
Central	305 (7.04)	809 (18.67)	
Southern	433 (9.99)	1165 (26.88)	
Eastern	49 (1.13)	135 (3.11)	
Residential city status			<0.0001 ^b^
Metropolis	3703 (85.44)	3055 (70.49)	
Satellite	148 (3.41)	291 (6.71)	
Rural	483 (11.14)	988 (22.80)	
Occupation			<0.0001 ^b^
Public servant	2460 (56.76)	2255 (52.03)	
Farmer	298 (6.88)	588 (13.57)	
Fisherman	42 (0.97)	86 (1.98)	
Other	1534 (35.39)	1405 (32.42)	
Ophthalmology visit times (times; Mean ± SD)	17.00 ± 28.70	6.53 ± 12.86	<0.0001 ^a^
Comorbid conditions			
Ocular allergic condition	204 (4.71)	8 (0.18)	<0.0001 ^b^
Asthma	312 (7.20)	313 (7.22)	0.9669 ^b^
Allergic rhinitis	152 (3.51)	140 (3.23)	0.4750 ^b^
Atopic dermatitis	62 (1.43)	79 (1.82)	0.1489 ^b^
Diabetes mellitus	310 (7.15)	428 (9.88)	<0.0001 ^b^
Chronic renal disease	75 (1.73)	89 (2.05)	0.2697 ^b^
Mitral valve prolapses	34 (0.78)	39 (0.90)	0.5567 ^b^

^a^ Paired *t*-test; ^b^ McNemar’s test. TWD, New Taiwan dollars; SD, standard deviation.

**Table 2:** Odds ratios and adjusted odds ratios for various sociodemographic factors and comorbid conditions with Fuchs’ endothelial dystrophy.

	Odds Ratio ^a^ (95% CI)	*p* Value	Adjusted Odds Ratio ^b^ (95% CI)	*p* Value
Sociodemographic factors				
Income				
<TWD 30,000	1.27 (0.90–1.78)	0.1704	1.92 (1.28–2.87)	0.0016
TWD 30,000–60,000	1.45 (1.04–2.04)	0.0302	1.72 (1.16–2.56)	0.0074
TWD 60,000–90,000	1.28 (0.88–1.87)	0.1934	1.40 (0.90–2.18)	0.1351
>TWD 90,000	1.00		1.00	
Geographical region of Taiwan				
Northern	4.80 (3.37–6.83)	<0.0001	5.33 (3.42–8.30)	<0.0001
Central	1.06 (0.73–1.53)	0.7635	1.13 (0.73–1.73)	0.5904
Southern	1.06 (0.74–1.54)	0.7418	1.06 (0.68–1.65)	0.7893
Eastern	1.00		1.00	
Residential city status				
Metropolis	2.53 (2.23–2.88)	<0.0001	0.97 (0.80–1.18)	0.7552
Satellite	0.96 (0.77–1.19)	0.7010	0.37 (0.28–0.50)	<0.0001
Rural	1.00		1.00	
Occupation				
Public servant	1.04 (0.94–1.15)	0.4298	1.02 (0.89–1.17)	0.7675
Farmer	0.43 (0.37–0.51)	<0.0001	0.92 (0.74–1.15)	0.4866
Fisherman	0.47 (0.32–0.68)	<0.0001	0.92 (0.58–1.46)	0.7197
Other	1.00		1.00	
Ophthalmology visit times	1.05 (1.05–1.06)	<0.0001	1.05 (1.05–1.06)	<0.0001
Comorbid conditions				
Ocular allergic condition	25.50 (12.58–51.68)	<0.0001	25.26 (11.24–56.77)	<0.0001
Asthma	1.00 (0.85–1.18)	0.9665	0.90 (0.73–1.12)	0.3406
Allergic rhinitis	1.09 (0.86–1.38)	0.4718	0.97 (0.71–1.32)	0.8477
Atopic dermatitis	0.78 (0.56–1.09)	0.1504	0.76 (0.49–1.18)	0.2267
Diabetes mellitus	0.68 (0.58–0.80)	<0.0001	0.52 (0.42–0.64)	<0.0001
Chronic renal disease	0.84 (0.62–1.15)	0.269	0.78 (0.52–1.18)	0.2372
Mitral valve prolapses	0.87 (0.55–1.38)	0.5532	0.63 (0.35–1.13)	0.1191

^a^ Odds ratio was obtained from a univariate logistic regression analysis; ^b^ adjusted odds ratio was calculated from a multivariable logistic regression model that was conditioned on age group, gender, and the year of index date. TWD, New Taiwan dollars; CI, confidence interval.

## Data Availability

All relevant data are within the paper.
